# Perls’ Stain Guidelines from the French-Speaking Cellular Hematology Group (GFHC)

**DOI:** 10.3390/diagnostics12071698

**Published:** 2022-07-12

**Authors:** Camille Lours, Laurane Cottin, Margaux Wiber, Valérie Andrieu, Véronique Baccini, Lucile Baseggio, Chantal Brouzes, Bernard Chatelain, Sylvie Daliphard, Odile Fenneteau, Franck Geneviève, Sandrine Girard, Vincent Leymarie, Karim Maloum, Jean-Baptiste Rieu, Gérard Sebahoun, Isabelle Sudaka, Xavier Troussard, Orianne Wagner-Ballon, Soraya Wuilleme, Valérie Bardet, Jean-François Lesesve

**Affiliations:** 1 Service d’Hématologie Biologique, Centre de Biologie et de Pathologie Est, Groupement Hospitalier Est, Hospices Civils de Lyon, 69500 Bron, France; sandrine.girard@chu-lyon.fr; 2 Laboratoire d’Hématologie, Centre Hospitalier Universitaire d’Angers, 49100 Angers, France; laurane.cottin@chu-angers.fr (L.C.); margaux.wiber@chu-angers.fr (M.W.); frgenevieve@chu-angers.fr (F.G.); 3 Département d’Hématologie et Immunologie, Groupement Hospitalier Bichat Claude Bernard, 75018 Paris, France; valerie.andrieu@aphp.fr; 4 Laboratoire d’Hématologie Immunologie, Centre Hospitalier Universitaire de Pointe-à-Pitre, 97159 Pointe-à-Pitre, France; veronique.baccini@chu-guadeloupe.fr; 5 Service d’Hématologie Biologique, Centre de Biologie et de Pathologie Sud, Hôpital Lyon Sud, Hospices Civils de Lyon, 69495 Pierre Bénite, France; lucile.baseggio@chu-lyon.fr; 6 Service d’Hématologie Biologique, Hôpital Necker, 75015 Paris, France; chantal.brouzes@aphp.fr; 7 Laboratoire d’Hématologie, Clinique Universitaire UCL de Mont-Godinne, 5530 Yvoir, Belgium; bernard.chatelain@gmail.com; 8 Laboratoire d’Hématologie, Centre Hospitalier Universitaire de Rouen, 76000 Rouen, France; sylvie.daliphard@chu-rouen.fr; 9 Laboratoire d’Hématologie, Hôpital Robert Debré, 75019 Paris, France; odile.fenneteau@aphp.fr; 10 Laboratoire d’Hématologie, Centre Hospitalier d’Avicenne, 93000 Bobigny, France; vincentleymarie@hotmail.fr; 11 Service d’Hématologie Biologique, Hôpital Pitié Salpêtrière, 75013 Paris, France; karim.maloum@aphp.fr; 12 Laboratoire d’Hématologie, Institut Universitaire du Cancer—Oncopôle, 31100 Toulouse, France; rieu.jb@chu-toulouse.fr; 13 Laboratoire d’Hématologie, APHM Hôpital Nord, 13005 Marseille, France; gerard.sebahoun@gmail.com; 14 Laboratoire d’Hématologie, Hôpital Pasteur, 06000 Nice, France; sudaka.i@chu-nice.fr; 15 Laboratoire d’Hématologie, Centre Hospitalier Universitaire de Caen, 14033 Caen, France; troussard-x@chu-caen.fr; 16 Département d’Hématologie et d’Immunologie Biologiques, Hôpital Universitaire Henri Mondor, 94000 Créteil, France; orianne.wagnerballon@aphp.fr; 17 Laboratoire d’Hématologie, Centre Hospitalier de Nantes, 44000 Nantes, France; soraya.wuilleme@chu-nantes.fr; 18 Service d’Hématologie-Immunologie-Transfusion, CHU Ambroise Paré, INSERM UMR 1184, AP-HP, Université Paris Saclay, 92100 Boulogne Billancourt, France; valerie.bardet@aphp.fr; 19 Laboratoire d’Hématologie, Centre Hospitalier Universitaire de Nancy, 54000 Nancy, France; jf.lesesve@chru-nancy.fr

**Keywords:** myelodysplastic syndromes, Perls’ stain, Prussian blue stain, cytochemistry, recommendations, bone marrow, iron store, iron staining, sideroblasts

## Abstract

In order to standardize cellular hematology practices, the French-speaking Cellular Hematology Group (Groupe Francophone d’Hématologie Cellulaire, GFHC) focused on Perls’ stain. A national survey was carried out, leading to the proposal of recommendations on insoluble iron detection and quantification in bone marrow. The criteria presented here met with a “strong professional agreement” and follow the suggestions of the World Health Organization’s classification of hematological malignancies.

## 1. Introduction

In addition to the standard May Grünwald–Giemsa stain of bone marrow (BM) aspiration films, Perls’ (or Prussian Blue) stain remains very useful for exploring the occurrence of iron in various hematological diseases. It aims to evaluate the BM iron stores and distribution. Perls’ stain is still recommended by the World Health Organization to classify hematopoietic malignancies, in addition to molecular, cytogenetic and immunophenotypic analysis development [1]. This staining is particularly useful to classify myelodysplastic syndromes (MDS) with or without ring sideroblasts (RS). How to perform and interpret this staining remains very heterogeneous around the world. With the ever-increasing development of the quality approach in laboratories and the need for accreditation, reference texts and standards are mandatory. To standardize practices, the GFHC proposes guidelines here about when and how to process insoluble iron detection in BM.

## 2. Methods

The French-speaking Cellular Hematology Group (GFHC) is the reference organization of the French Society of Hematology (Société Française d’Hématologie, SFH) for all concerns about hematological morphology. The following recommendations come from a large practice survey conducted by the GFHC between 2018 and 2019 among French-speaking specialists in laboratory medicine. A unique online survey was sent to the 270 specialists among the laboratory members of the GFHC (from France, Belgium, Switzerland, Luxemburg, Canada and French-speaking African countries). As practices are usually homogeneous in one given center, we asked for a single response per center. A total of 95 participants completed the study. In all, 54% of the responses were from university hospitals from France and other countries, 37% from general hospitals, 6% from private laboratories and 3% from cancer centers, which is representative of all of the GFHC members. The answers were reviewed, discussed and compared to those given from the international literature.

## 3. Guidelines

### 3.1. Indications

Perls’ stain is used to evaluate extracellular and macrophagic iron stored in BM. It also shows insoluble iron in red blood cells (siderocytes) and erythroblasts (sideroblasts) [2]. Siderocytes are medullar red blood cells containing precipitates of iron complexed with proteins. They are usually absent in healthy patients. Sideroblasts are erythroblasts with endosomes filled with iron for heme synthesis. While a few sideroblasts are normally present in bone marrow, ring sideroblasts (RS) are found only in pathological conditions; they correspond to erythroblasts with abnormal iron accumulation in the mitochondria, forming a ring around the nucleus. The presence of RS usually signifies that erythropoiesis is ineffective.

The first indication of Perls’ stain is to detect abnormal iron accumulation in erythroblasts and erythrocytes (or other cells) and to quantify ring sideroblasts if present.

The WHO classification of hematopoietic malignancies defines two categories of myeloid neoplasms by the presence of RS: myelodysplastic syndromes with RS (MDS-RS) and myelodysplastic/myeloproliferative neoplasm with ring sideroblasts and thrombocytosis (MDS/MPN-RS-T) [1]:

MDS-RS: MDS characterized by cytopenias, morphological dysplasia and ring sideroblasts constituting ≥ 15% of the bone marrow erythroid precursors, or ring sideroblasts ≥ 5% of the bone marrow erythroid precursors associated with *SF3B1* mutation. The blasts account for <5% of the nucleated bone marrow cells.

MDS/MPN-RS-T: A subtype of MDS/MPN characterized by thrombocytosis ≥ 450 G/L associated with ring sideroblasts accounting for ≥15% of the bone marrow erythroid precursors, dyserythropoiesis and <5% blasts in bone marrow. The rate of RS required for the diagnosis of MDS/MPN-RS-T is not altered by the presence or the absence of *SF3B1* mutations.

Secondary causes of ring sideroblasts must be excluded in both cases.

The biological features of the two entities are summarized in Table 1 (from Patnaik et al., AJH 2021 [3]).

The assessment of iron repartition has to be completed every time that a diagnosis of MDS is suspected [4]. Above all, the ring sideroblasts quantification is essential to properly classify MDS [1,4]. It should also be executed to explore thrombocytosis (particularly in association with anemia or dyserythropoiesis) to differentiate essential thrombocythemia and MDS/MPN-RS-T. Note that ~50% of MDS/MPN-RS-T carry the JAK2 V617F mutation [3].

Interestingly, the cost of Perls’ stain is very low, compared to molecular tests (6.75€ versus about 1500€ for Next Generation Sequencing including *SF3B1* mutation screening): that is why the preliminary evaluation of the ring sideroblasts percentage is recommended, instead of systematic molecular screening of *SF3B1*.

The GFHC highlights the importance of Perls’ stain to evaluate iron repartition in cases of unexplained anemia and BM dysplasia. Overall, it is useful to evaluate the iron stores in BM (in association with other means of iron exploration), and to detect sideroblasts in various pathologies (such as inflammatory syndrome or lead poisoning).

### 3.2. Procedure

The addition of potassium ferrocyanide on a BM film allows iron detection by forming colored precipitates with ferric ions (Fe^3+^): the reaction produces insoluble blue-colored ferric ferrocyanide. It must be completed in an acidic environment to break the atomic bonds between the iron and proteins, such as hemosiderin. Ferritin contains soluble iron and so is not detectable by Perls’ stain, nor is ferrous iron (Fe^2+^) in hemoglobin. Red counterstaining is recommended to enhance the cells’ identification (to help to differentiate the lymphocytes and erythroblasts, in particular) [5] and iron granules count. The rinsing can be completed with distilled or tap water without a significant difference.

Laboratories can use homemade procedures or commercial kits in conformity with the local regulations; the GFHC proposes a quick and robust protocol below (Section 4, below).

BM aspiration should be completed following the local recommendations. Note that the GFHC previously wrote recommendations for BM sampling and examination [6]. The transport and storage of fresh BM films are completed at room temperature before staining.

The GFHC recommends Perls’ stain realization at least within two weeks, which remains compatible with the usual delay for a global interpretation of the BM samples. A later realization can be completed, as it would not have any significant consequence on the ring sideroblasts count. A positive control is required in each run, such as a BM smear with a raised amount of extracellular or macrophagic iron.

### 3.3. Interpretation

Perls’ stain has to be executed in addition to MGG-stained blood and BM smears’ interpretation. The smear reading should be executed by well-trained and qualified operators. A systematic double counting is not necessary, except in the case of the presence of ring sideroblasts near the thresholds of 5% and 15% of nucleated erythroid cells, or in the case of discordance of interpretation with the MGG smear (doubtful dysplasia, in particular).

A count of 200 erythroblasts is recommended, especially when the ring sideroblast percentages are close to 5 or 15%, however 100 can be accepted, e.g., in cases of low cellularity. The international authorities (including the International Working Group on Morphology of Myelodysplastic Syndrome (IWGM-MDS) guidelines) recommend that all of the erythroblasts should be considered: proerythroblasts; basophilic erythroblasts; polychromatophilic erythroblasts; and orthochromatic erythroblasts. However, it is worth noting that the iron in proerythroblasts and basophilic erythroblasts accumulates in micelles too small to be easily detected by light microscopy. There is, thus, a risk of moderate overestimation of global sideroblastosis if the proerythroblasts are not counted. Moreover, the recognition of the proerythroblasts may be more difficult if the counterstaining is not optimal.

The issue of the percentage’s confidence interval in case of a small percentage is well known: for 100 erythroblasts evaluated, the 95% confidence interval of the observed value 5% varies from 2 to 11%, and for 200 erythroblasts, it varies from 2 to 9%. For an observed value of 15%, for 100 erythroblasts evaluated, the 95% confidence interval varies from 9 to 23%, and for 200 erythroblasts, it varies from 10 to 21% [7]. It is therefore preferable to establish a criticality analysis for the results close to 5 and 15% of sideroblasts, requiring repeatability/reproducibility studies. For example, these can be performed during an inter-laboratory control.

The precise definition of the sideroblast types has been detailed by the IWGM-MDS [8]:

- Type 0: no visible granules;
- Type 1: less than five granules in the cytoplasm;
- Type 2: five or more granules but not peri-nuclear;
- Ring sideroblasts (or type 3): five or more granules in a peri-nuclear position, encircling one-third or more of the circumference of the nucleus.

Only the ring sideroblasts percentage must be taken into account for the WHO Classification.

**Figure 1:**
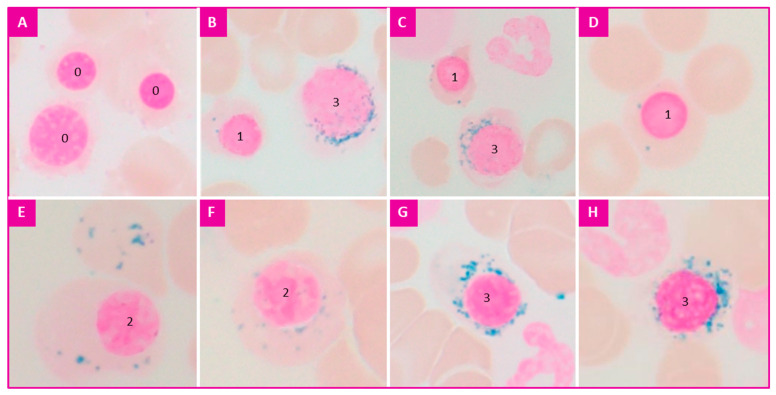
The different types of sideroblasts: (**A**): Three type 0 sideroblasts, (**B**,**C**): One type 1 and one Ring Sideroblast (or type 3), (**D**): One type 1 sideroblast, (**E**,**F**): One type 2 sideroblast, (**G**,**H**): One Ring Sideroblast.

Typical examples are illustrated in Figure 1.

The observation of the sideroblasts is optimal at a high magnification (×100). The detection of type 1 requires varying the micrometer screw of the microscope. The detection of type 2 and 3 can be completed at a medium magnification (×50) (Figure 1).

The overall interpretation can be as follows:

1. Siderocytes are normally absent;
2. In the case of sideroblastic anemia, several sideroblasts can be observed per field (obvious, and most often at least five per field at ×100 magnification);
3. In the absence of iron deficiency, a few iron-containing macrophages are always observed (at least one macrophage out of five is stained);
4. Reference values have been proposed for sideroblasts:
  - type 0: 60–90%,
  - type 1: 10–40%,
  - type 2: <1% (a count of less than one type 2 sideroblast among 100 erythroblasts is considered insignificant).
  - type 3: <1% (a count of less than one ring sideroblast among 100 erythroblasts is considered insignificant).

The following interpretive comments can be suggested:

- Absence of pathological sideroblastosis;
- Numerous sideroblasts with some ring sideroblasts between 5 and 14%. To be completed by a *SF3B1* gene mutation research;
- Presence of pathological sideroblasts, with ring sideroblasts ≥ 15%. In favor of MDS with ring sideroblasts (in the absence of excess blasts and Auer rods, and diagnostic criteria for myelodysplastic/myeloproliferative neoplasm with ring sideroblasts and thrombocytosis).

Even when the observation of 100 erythroblasts is made difficult, either by a low cellularity BM smear or an erythroblastopenia, useful information could still be available to guide the diagnosis. Conclusions, such as “no ring sideroblasts were observed among the rare erythroblasts observed” or, conversely “the rare erythroblasts observed are mainly ring sideroblasts”, should be proposed.

**Figure 2:**
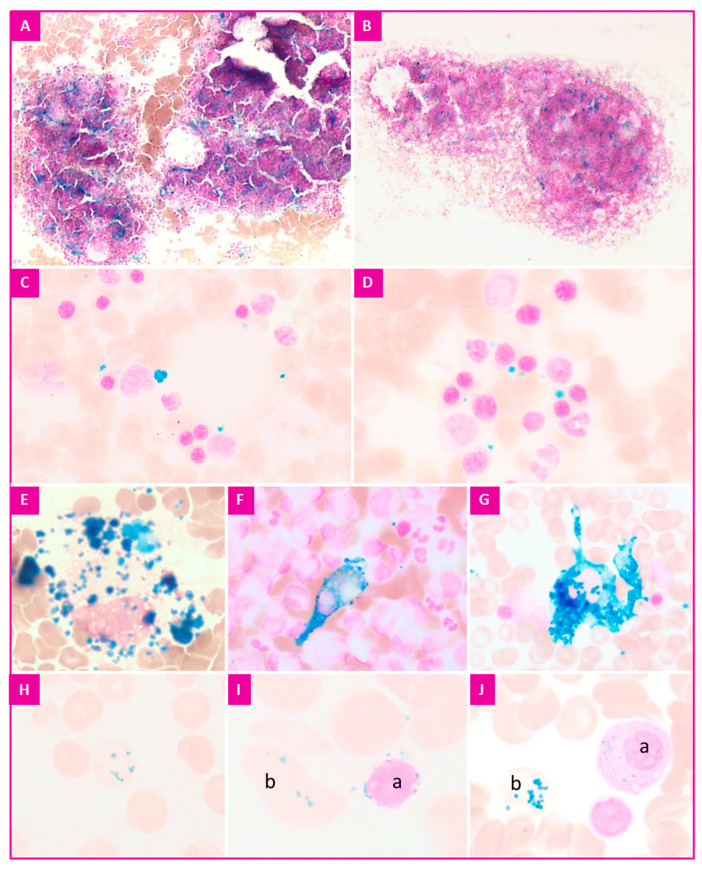
Prussian blue reaction of erythroid precursors, hematoxylin counterstain. (**A**,**B**) medullar iron content evaluation; (**C**,**D**) extracellular iron deposits; (**E**–**G**) macrophages; (**H**) siderocyte; (**I**) sideroblast (a) and siderocyte (b); and (**J**) hemosiderin-containing plasma cell (a) and one siderocyte (b).

Perls’ stain also provides three other pieces of information (Figure 2):

1. The macrophagic iron content;
2. The presence of red blood cells containing precipitates of iron complexed with proteins (Pappenheimer bodies in MGG staining, siderocytes in Perls’ stain);
3. The presence or absence of extracellular iron [9].

However, the evaluation of medullary iron stores through BM biopsy observed by anatomopathologists remains the reference method.

In the absence of abnormal sideroblastosis, the iron load in the macrophages directly reflects the iron use in erythroblasts for the hemoglobin synthesis.

The percentage of the type 1 and 2 sideroblasts is significantly decreased in both inflammatory and iron-deficiency anemias. The macrophage iron load is also reduced or absent in iron deficiency, and remains normal or increases in inflammation.

When a bone marrow biopsy is performed to explore unexplained anemia, an evaluation of the macrophagic iron store is always useful to discriminate between inflammatory and deficiency mechanisms.

A global interpretation is useful, taking into account the presence of siderocytes, whole sideroblasts, normal or not, and the global load of the macrophages in iron, decreased, normal or increased, in order to orientate towards an iron deficiency, an inflammatory syndrome or an iron overload.

The following standardized comments can be suggested:

- Overall iron content of macrophages and iron distribution in erythroblasts are normal. In favor of undisturbed iron utilization in erythroblasts for hemoglobin synthesis;
- Decreased overall iron content of macrophages. Normal/decreased sideroblastosis. In favor of a deficit of iron in erythroblasts for hemoglobin synthesis. To be explored;
- Increased global iron content of macrophages. Sideroblastosis in the levels normally observed. In favor of an inflammatory process, the cause of the inflammation should be investigated.

Ring sideroblasts may be observed without relation to MDS, acute leukemia with dysplasia or *SF3B1* gene mutation. There is no artifact but the origin may be iatrogenic/toxic or in the context of other pathologies [10]:

- Benzene, chemotherapy (Melphalan), antibiotics (Chloramphenicol, Linezolid), anti-tuberculosis drugs (Isoniazid, Pyrazinamide, Cycloserine);
- Thalassemias, inflammatory syndromes, copper deficiency (possibly secondary to excessive ingestion of zinc), liver diseases (Wilson’s Disease), chronic alcoholism, lead poisoning, arsenic poisoning, severe undernutrition, deficiency of vitamins B1, B6, B9, B12, poly-transfusion, thiamine-sensitive megaloblastic anemias with mutation of the *SLC19* gene, Wolfram syndrome (DIDMOAD: ‘diabetes insipidus, diabetes mellitus, optic atrophy and deafness’);
- Constitutional sideroblastic anemias, mitochondrial cytopathies (Pearson syndrome).

For information, iron precipitates can sometimes be observed in plasma cells in the case of overload (hemochromatosis, chronic alcoholism, multiple transfusions) [11].

The observation of siderocytes is always abnormal: a search for hemolytic anemia, MDS or splenectomy should be a priority.

### 3.4. Reporting Arrangements

It is recommended to mention the percentages of the different types of sideroblasts (0, 1, 2, 3 and ring sideroblasts), as well as an overview of the estimation of the siderocytes (presence/absence) and the medullary iron reserves (“absence/presence/increase” of iron content in macrophages).

The report can or cannot be attached to the BM aspiration report. An interpretation and an indication of the potential diagnosis should be included in at least one of the two reports. A phone call with the results would lead to a dialogue with the physician to discuss the assessment and diagnosis, but communicating the result is not considered an emergency.

### 3.5. Conservation

Perls’ stained BM smears should be kept with the corresponding patients’ MGG-stained BM smears. The duration of the storage of BM smears should comply with national regulatory guidelines. When these are not available, the BM slides should be stored dust free at room temperature for at least 20 years.

### 3.6. Quality Controls

The frequent use of accurate quality control is recommended to ensure the reliability of reported results, and therefore optimum patient care. It is advisable to perform Internal Quality Control (IQC) from patients’ BM smears at least once a year. This IQC should include at least one smear with few sideroblasts and another with excessive RS ≥15%. Where possible, annual participation in an External Quality Assessment (EQA) is required, particularly in the case of an accreditation process (one is in use in France, four times a year). All of the staff, technical and medical, involved in reading Perls’ stains should take part in these controls.

## 4. Technical Protocol

**Figure 3:**
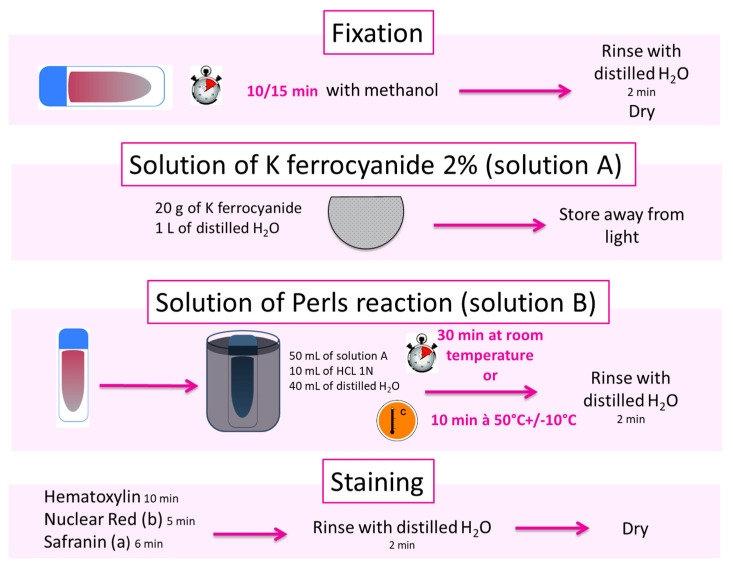
Technical protocol of Perls’ Stain.

The following protocol (Figure 3) aims to guide users with a procedure and reagents independent of the commercial kits. The technique is fast (less than an hour) and robust. The toxicity is reduced. However, it is necessary to work with a lab coat and gloves, and possibly under a chemical fume hood.

**Equipment:**

- Non-specific laboratory equipment: glass dishes, test tubes, pipettes, staining trays, smear racks, timer;
- Adjustable water bath at 50 °C.

**Reagents:**

- Hydrochloric acid 1 N stored at room temperature (secure cabinet);
- Hematoxylin of Harris, of Mayer or Nuclear test Red;
- Absolute methanol stored at room temperature (secure cabinet);
- Potassium ferrocyanide (potassium hexacyanoferrate or ferrocyanide trihydrate) stored at room temperature (secure cabinet);
- Aluminum sulfate 98% (optionally);
- Nuclear red or safranin (optionally).

**Reagents preparation:**

Solution A:

Aqueous solution (2%) of potassium ferrocyanide: 20 g in 1 L of distilled water, store at room temperature away from light.

Solution B:

60 mL of solution A + 12 mL of HCl 1N + 48 mL of distilled water.

Nuclear red:

5 g of aluminum sulfate to dissolve in 100 mL of water, then add 0.2 g of nuclear red. Shake for 8 to 12 h then store at +4 °C away from light.

**Smears:**

Bone marrow or blood, fresh or frozen (several months), old smears possible.

**Modus operandi:**

- Fix patient’s BM or blood smears and the control smear for 10 min by covering them with methanol;
- Rinse with distilled water and then dry;
- Put the smears in a tank equipped with a smear holder;
- Put solution B in the tank containing the smears and place it in a water bath at 50 °C for 10 min;
- Rinse with distilled water and then dry;
- Counterstain by covering with hematoxylin for 10 min or with nuclear red for 5 min or with safranin (6 min);
- Rinse with distilled water and then dry.

**Reading**

Refer to the advice mentioned in Section 3.3.

Observe extracellular and intracellular iron (macrophages, red blood cells, erythroblasts).

Establish a formula on 100 erythroblasts and classify the erythroblasts without granules (type 0), with less than four granules (type 1), with many granules but not arranged in ring (type 2) and ring sideroblasts (type 3).

## 5. Conclusions

Despite the development of next-generation sequencing approaches, basic morphological evaluation is essential to assess a dysplastic state and remains the cornerstone of MDS diagnosis. In this context, Perls’ stain for the quantification of ring sideroblasts remains necessary for the categorization of MDS, according to WHO 2017 recommendations [1]. This staining can also be very informative in many other situations to evaluate medullar iron repartition.

## Figures and Tables

**Table 1:** Biological features of MDS-RS and MDS/MPN-RS-T (BM: Bone Marrow; PB: Peripheral Blood; SLD: Single Lineage Dysplasia; MLD: Multilineage Dysplasia; MPN: Myeloproliferative Neoplasms).

Variable	MDS-RS	MDS/MPN-RS-T
Blood and bone marrow findings	Presence of cytopenia, morphological dysplasia and BM RS ≥ 15%, or RS ≥ 5% in the presence of SF3B1 mutations.Can have single lineage or multi-lineage dysplasia (MDS-RS-SLD and MDS-RS-MLD). <1% PB blasts, <5% BM blasts and no Auer rods.	Anemia with erythroid lineage dysplasia, with or without multi-lineage dysplasia, ≥15%BM RS. <1% PB Blasts, <5% BM blasts and no Auer rods. Megakaryocytic hyperplasia with morphological features similar to those seen in BCR-ABL1 negative MPN.
Platelet counts	Normal to decreased	Persistent thrombocytosis ≥ 450 G/L
BM RS (%)	BM RS ≥ 15% or BM RS≥5% in the presence of SF3B1 mutations.	BM RS ≥ 15% regardless of the presence or absence of SF3B1 mutations.
Cytogenetic/Molecular categories that exclude a diagnosis	Del(5q)-MDS	BCR-ABL1 PDGFRA, PDGFRB, FGFR1 PCM1-JAK2 Del(5q) t(3;3)(q21;q26), inv(3)(q21;q26)
Frequency of cytogenetic abnormalities	20% MDS-RS-SLD 50% MDS-RS-MLD	20%
Molecular signature	SF3B1—80% TET2—30% DNMT3A—20% ASXL1—15% RUNX1—10% TP53—5%	SF3B1—80% JAK2 V617F—50% TET2—20% DNMT3A—15% ASXL—15% SETBP1—10% MPL—5% SH2B3—5%
